# Mobile phone support to sustain exclusive breastfeeding in the community after hospital delivery and counseling: a quasi-experimental study

**DOI:** 10.1186/s13006-020-00258-z

**Published:** 2020-03-04

**Authors:** Iftia Jerin, Monira Akter, Khurshid Talukder, Muhammad Qudrat e Khuda Talukder, Mohammad Abdur Rahman

**Affiliations:** 1 Centre for Woman and Child Health (CWCH), Savar, Dhaka, 1349 Bangladesh; 2 Nutrition International, Gulshan, Dhaka, 1212 Bangladesh; 3 Department of Public Health and Informatics, Jahangirnagar University, Savar, Dhaka, 1342 Bangladesh

**Keywords:** Exclusive breastfeeding, Bangladesh, Breastfeeding initiation, Counseling, Mobile phone

## Abstract

**Background:**

Rapid increases in hospital and cesarean deliveries threaten an already falling exclusive breastfeeding rate (EBR) in Bangladesh. There is neither a sustained Baby-Friendly Hospital Initiative (BFHI) nor any community support for breastfeeding mothers. Our aim was to find out whether breastfeeding support after hospital delivery and subsequently by mobile phone at home is effective in improving EBR in infants under six**-**months of age.

**Methods:**

A quasi-experimental study was carried out in 2010 at the Centre for Woman and Child Health (CWCH), Savar, Bangladesh. A total of 129 mothers delivered at CWCH were recruited in pre**-**intervention phase and their infants followed up between 0 and 5 months of age in the community for exclusive breastfeeding (EBF), anthropometry and illness. An intervention package was then implemented with postpartum support for first hour breastfeeding initiation, correction of position and attachment and face-to-face counseling in hospital followed by mobile phone support by two trained Research Assistants once every 15 days after discharge up to six months of age. During the intervention phase, 164 pregnant women delivered at CWCH were recruited and followed up as in the pre-intervention phase.

**Results:**

In the pre-intervention phase among 114 infants, 66 (58%) were found to be exclusively breastfed. In the intervention phase among 151 infants, 118 (78%) were exclusively breastfed (*p* = 0.000). In the pre-intervention phase EBR at less than one month and five months were 85 and 42% as in the intervention phase these EBR were 89 and 71% respectively. Wasting (weight**-**for**-**height Z**-**score < **−** 2.00), stunting (height**-**for**-**age Z**-**score < **−** 2.00), and underweight (weight**-**for**-**age Z**-**score < **−** 2.00) was 17 (15%), 7 (6%), and 14 (13%) respectively in the pre-intervention phase. In the intervention phase wasting, stunting, and underweight was 16 (11%), 16 (11%), and 15 (10%) respectively. Therefore, there was no statistically significant differences in nutritional status of the infants in the two phases. There was also no significant differences in child morbidity (pneumonia and diarrhea) between the two phases.

**Conclusion:**

A combination of hospital support and mobile phone counseling in the community sustained higher rates of EBF in the community after hospital delivery.

## Background

Breastfeeding is the best means of optimal nutrition, growth, development and survival for infants and young children [1, 2]. It is now well established that suboptimal breastfeeding is associated with increased risks of malnutrition [3–5], morbidity [6, 7] and mortality [8–11] in children, especially in low- and middle-income countries.

In Bangladesh, there has been a fivefold increase in facility-based deliveries from 9% in 2001 to 47% in 2016 [12]. Since 1992, 499 out of 670 maternity units in the country had achieved baby friendly status under the Baby-Friendly Hospital Initiative (BFHI) but frequent turnover of trained health personnel, lack of refresher training and weak implementation of the Breast Milk Substitute (BMS) Act were major contributing factors for significant deterioration [13, 14] in breastfeeding promotion. This was further compounded by non-existent support for lactating mothers in the community after discharge. All these threaten an already declining annual exclusive breastfeeding rate (EBR – in previous 24 h) in infants below six months of age of (64%, in 2011 and 65% in 2014) and this decline is further compounded by an undesirable high rate of cesarean sections, 65% of all facility deliveries in 2016 [12]. In private hospitals the rate was 83%, in Non-Government Organization (NGO) hospitals 39% and, in public hospitals 35% [12]. A recent Bangladeshi study [15] as well as a systematic review [16] has found strong evidence that cesarean section can impede exclusive breastfeeding (EBF).

A systematic review [17] of global breastfeeding programs showed that despite poor overall study quality, structured programs had an impact on breastfeeding performance. A Cochrane review [18] that analyzed trials of support for breastfeeding showed an increased EBR at six**-**months. Support was more effective when it was in the setting of antenatal and postnatal care, took place during planned points of contact, and adapted to local needs. Support was also more successful in populations with high initiation rates and when it was provided through interpersonal communication.

Another Cochrane review [19], which documented results from seven trials of breastfeeding support by telephone, suggested that such support could increase breastfeeding duration. This review noted that providing telephone support for breastfeeding increased EBF at six months. A study in Nigeria [20] showed that breastfeeding related text messages were effective in improving EBR at six months. Another study in India [21] showed that mobile phone and text message interventions for lactation support improved EBF measured at multiple points of time. Together, these studies suggest that breastfeeding messaging or support offered by mobile phone could potentially be a low cost strategy for improving breastfeeding practices in a variety of settings.

**Fig. 1:**
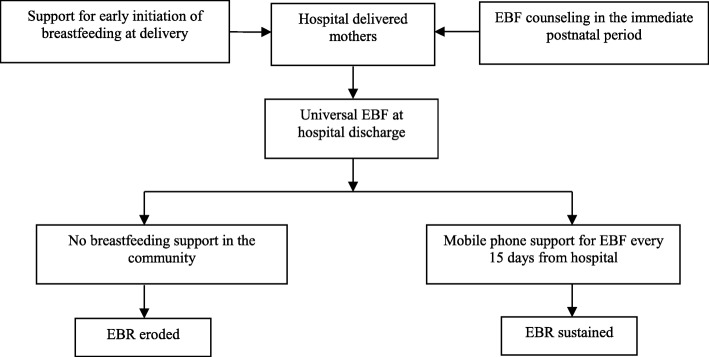
Theoretical framework for sustaining EBF in the Community by mobile phone promotion

Our theoretical framework for this study is that, although high rates of EBF are possible by promotion in hospitals at delivery and in the immediate postnatal period by trained personnel, these rates are rapidly eroded due to lack of structured support in the community [22, 23]. We hypothesize that facility based trained personnel can continue to provide outreach breastfeeding counseling services in the community by mobile phone and sustain EBF effectively (Fig. 1).

Based on the above, we carried out a quasi**-**experimental study with the objective of evaluating the impact on EBR of facility based breastfeeding promotion of delivering mothers followed by fortnightly mobile phone breastfeeding counseling calls at home for six months after discharge. Other outcomes of interest were infant illness and anthropometry.

This quasi**-**experimental study was designed to assess whether a package of face**-**to**-**face and phone based breastfeeding promotion and support leads to increase in EBR in infants below six months of age following hospital delivery. This is important in a low middle**-**income country like Bangladesh where hospital-based deliveries and cesarean sections are increasing rapidly.

## Methods

### Design

In this quasi-experimental study EBR was assessed in young infants aged 0 and 180 days of age post-delivery in the community in serially recruited pre and post-intervention phases of postnatal mothers without randomization to intervention.

### Setting

Mother**-**infant pairs delivered at the Centre for Woman and Child Health (CWCH), a secondary level health facility 28 km to the north**-**west of Dhaka Metropolitan Area in Bangladesh, were studied immediately after delivery as well as in the community after discharge between April and December 2010. Almost all infants delivered at this hospital are breastfed within one hour of delivery. This coupled with 93% breastfeeding prevalence in infants at 9–11 months nationally [24] ensured that recruitment took place in no more than three months of each phase of the study.

### Intervention

A curriculum was developed by a participatory method involving the hospital’s research team, lactation management team, obstetrician/gynecologists (OB/GYN), pediatric physicians and maternity registered nurses (RN). The curriculum also incorporated features from international literature on Infant and Young Child Feeding (IYCF) training for health personnel [25–30]. Initially a one-day orientation training session was conducted for CWCH physicians from the OB/GYN and Pediatric departments and RNs by a nutritionist and lactation consultant, both of whom were female. They were given in-depth understanding of benefits of first hour initiation of breastfeeding; disadvantages of late initiation of breastfeeding, techniques of first hour initiation of breastfeeding, in both normal and cesarean deliveries, barriers to initiation of breastfeeding in the first hour and how to overcome those barriers.

Five maternity RN from CWCH and two Research Assistants (RA) then received a three**-**day training course on breastfeeding, which included basic information on breastfeeding, counseling and support techniques as well as practical sessions on observation and assessment of breastfeeding and counseling in the hospital. Topics covered included, (i) importance and basic features of breastfeeding, (ii) techniques of breastfeeding, (iii) milk expression, (iv) common problems of the breasts during breastfeeding and their solution and (v) counseling skills on breastfeeding with emphasis on listening, learning, building confidence and giving support, specially the avoidance of judging words to prevent maternal guilt and blame. Training methods included lectures, hands-on demonstration, and practical exercises with real-life postpartum breastfeeding mothers in hospital and video-guided lessons. The two female trainers observed each trainee RN while assessing a new breastfeeding mother, using a structured checklist.

Trained maternity personnel (RNs under guidance from the lactation consultant and CWCH physicians) ensured early initiation of breastfeeding immediately after birth for all mother-infant pairs. For intervention mothers, RNs also assessed position and attachment of the infant within 24 h and corrected any problem detected by repeated face-to-face tailor-made support of both mothers and attending relatives. In addition, RNs also promoted appropriate IYCF practices in a counseling class for intervention mothers and their relatives during their stay in hospital. This class covered advantages of breastfeeding, dangers of formula feeding, how to ensure EBF up to six months of age, advantages of EBF, common breast problems during breastfeeding and their solutions, family support for EBF, how to express breast milk during infant’s illness and how to start complementary food after six months. These classes were conducted within 48 h of birth for cesarean deliveries and 24 h for vaginal deliveries.

After discharge from hospital, intervention mother**-**infant pairs were followed up (Table 1) by mobile phone counseling calls every 15 days by two trained RAs up to six months of age. These calls followed a structured interview protocol. During the calls, which lasted for approximately 15 min, the mother was asked what she was feeding her infant, confirming that not even water was given to those infants whose mothers said that they were feeding nothing but human milk. If the response was EBF, she congratulated the mother and advised her to continue this good practice up to six months of age, avoiding even feeding water. If the mother was not exclusively breastfeeding, then the RA asked her why she was not. If the reason given was inadequate human milk then the RA asked her about the infant’s urine output and weight gain, emphasizing that colorless urine passed more than six times a day and weight gain were signs that human milk was adequate. She also re**-**emphasized the importance of position and attachment, which had been demonstrated during the hospital admission. Finally, she reiterated the advantages of EBF and the dangers of formula feeding. If the mother still expressed doubts about her ability to exclusively breastfeed, the RA advised consultation with the lactation consultant in the CWCH.

**Table 1:** Mobile phone support during community follow up by Research Assistant

Problem	Solution provided
Inadequate human milk	Signs of getting enough human milk – • urine passed 6–8 times in 24 h • urine is not yellow but is clear • weight gain 500 g per month
Crying baby	• Crying is the baby’s only language • Value of a full breastfeed - foremilk and hindmilk • Proper breastfeeding position and attachment
Baby does not take mother’s breast	Proper breastfeeding position and attachment
Breastfeeding is hard and mother doesn’t have the energy	• Benefits of human milk for child • Benefits of human milk for mother • Value of a full breastfeed - foremilk and hindmilk
Working mother	• Human milk expression • Human milk storage • Feeding human milk with a spoon/cup • Disadvantages and dangers of formula/processed milk
Different types of breast problems like engorgement, blocked duct, sore nipple, inverted nipple	Consultation with the lactation consultant at CWCH

### Sample

Mothers were recruited in this study by a Research Assistant (RA) who collected data regarding patient identity, delivery and postnatal care from the postnatal ward, usually the day after delivery at CWCH. Eligibility criteria included all mothers who delivered in the hospital, intended to breastfeed within one hour of birth, planned to breastfeed infants exclusively, were resident in the study area for six months or more after delivery, and owned a mobile phone. Infants with apparent health problems, prematurity and extremely low birthweight (< 1000 g), mothers with complicated cesarean sections and compromised maternal health were excluded because of the possible inability of the mother or infant to initiate breastfeeding.

Mothers recruited in the pre-intervention phase experienced routine breastfeeding support care in hospital immediately after delivery. The hospital did not have baby friendly status but maternity personnel would put the infant to breast within one hour of birth provided maternal and newborn clinical conditions (such as perinatal asphyxia) allowed this. There was no pro-active promotion and support of breastfeeding practices but problem-oriented support was provided to mothers for breastfeeding if it was requested. Examples of support provided would be assessment and correction of position and attachment, extra help for mothers with engorged breasts or sore nipples or for those who delivered by cesarean section.

### Data measures

Exclusive breastfeeding rate is the proportion of under six**-**month old infants in a population who have been fed nothing but human milk in the 24 h prior to the interview. Accessing this information from uniformly distributed infants of different ages is not easy, especially if there is time constraint in data collection. We somewhat mitigated this difficulty by interviewing mothers in the community in reverse order to the time of discharge from hospital after delivery, as described in the data collection section below. Our questionnaire included a question asking the mother what she was currently feeding her infant. If she said only human milk then follow up questions were asked specifically about whether water, infant formula, animal milk or any other food was also given. This line of questioning has previously been validated in Bangladesh by stable isotope studies on infants whose mothers claimed that they had fed their infants exclusively on human milk for the previous 24 h [31].

### Data collection

Pre**-**intervention data were collected for the first six months (April to September, 2010) of the study period by the RAs both in hospital and community using a structured questionnaire. During hospital data collection, delivery procedures and breastfeeding initiation practices in the labor room/operation theatre were documented. Neonatal details such as age, gender, gestational age, respiration, Apgar score, mode of delivery, any problems during birth and maternal medical details such as history of mother’s medical and obstetric problems and other delivery related information were collected from medical records. Mothers were interviewed regarding sociodemographic characteristics, antenatal care, contraceptive use and perception about EBF. All information was collected within 48 h of delivery. Anthropometric measurement (height and weight) of mothers was done before delivery and of infants immediately after birth.

In order to assess EBR in the community infants enrolled in the pre**-**intervention phase were followed in “reverse order” in the community so that the last hospital recruited infants were visited first and the first recruited infants was visited last. In this way, we recruited a range of infants from seven to180 days of age. During home visits, mothers were asked about EBF and infant morbidity. Infant’s weight and height were measured using a portable electronic baby scale accurate to 0.01 kg and a portable height scale accurate to one millimeter.

During the intervention phase (July to December, 2010), hospital data were collected for three months from recruited mother**-**infant pairs by using the same procedures and the same questionnaire as in the pre**-**intervention recruitment. After hospital discharge, these infants were followed in the community at less than six months of age by using the same “reverse order” mechanism and questionnaire as in the pre**-**intervention phase.

### Data analysis

In the Bangladesh Demographic and Health Survey (BDHS) 2007 report [32], EBR was reported as 47% and we assumed that after intervention there would be a 25% point increase in EBR among intervention infants from this pre**-**intervention figure. This estimate was based on a randomized control trial [33], which showed EBF at five months was 70% after a community-based promotion of breastfeeding. We wanted to increase our EBR from an assumed rate of 47% in our study area to 70% (i.e. a 25% point increase). It was estimated that the sample size needed to detect differences between intervention and pre**-**intervention phases (with 0.05 alpha and 80% power) would be 103 mother**-**infant pairs in each phase, which was enhanced to 129 by expecting 25% lost to follow up in the study area. Data entry and validation was done using Epi**-**info and analysis was done using Statistical Package for the Social Sciences (SPSS) software. We compared sociodemographic parameters, delivery information, maternal factors, infant feeding practices, anthropometry [25] and illness data between the pre**-**intervention and intervention mother**-**infant pairs using the chi**-**square test. Mean number of family members and birthweight were compared by using Independent Samples *t***-**test. Our main comparison of interest was EBR in the pre**-**intervention and intervention infants under six months of age.

## Results

**Fig. 2:**
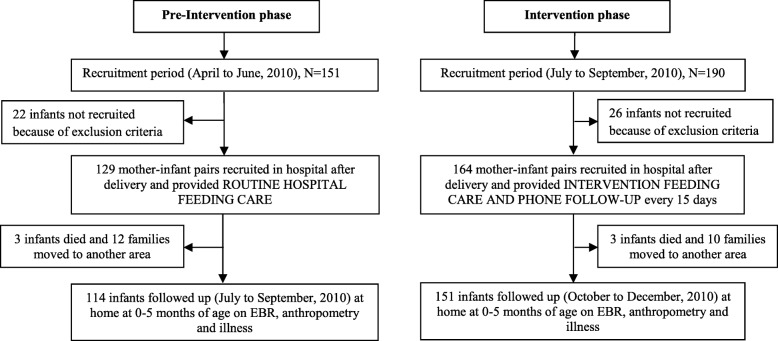
Flow diagram of participants

Mother**-**infant pairs were recruited and interviewed in the pre-intervention and intervention phases as shown in Fig. 2. Of the 151 deliveries that took place at CWCH in the pre-intervention recruitment phase (April**–**June 2010), 129 mothers were recruited for the study, 22 mother**-**infant pairs not fulfilling inclusion criteria. Out of the 129 mothers enrolled, 114 were found at the time of community follow up and therefore were included in this analysis. Of the 15 lost to follow up, three infants had died and another 12 mothers had moved back to their village homes. During the intervention recruitment phase (July**–**September 2010), there were 190 deliveries in the study hospital of which 164 mothers were enrolled, with 26 mother**-**infant pairs not recruited because they did not meet inclusion criteria. At the time of community follow up, 151 mother**-**infant pairs were located and these are the pairs included in the analysis. Among 13 families not located in follow up, three infants had died and 10 mothers had moved back to their village homes.

All intervention phase mothers received a breastfeeding promotion session in a class setting. They also all received a first assessment and correction (if needed) of position and attachment, followed by further few support sessions (if needed) for a select few. In the community, the number of fortnightly phone calls received by the mothers depended on the age of the baby by the end of the study. So, if the baby was only two weeks old (near the end of the study) the mother will have received only one phone call. Those mothers whose babies were almost six months of age at the end of the study will have received a maximum of 12 phone calls.

**Fig. 3:**
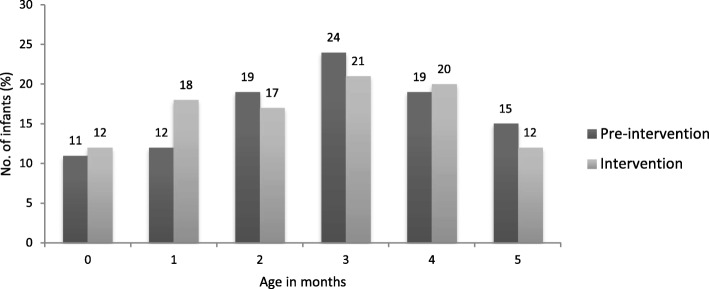
Age distribution of infants followed up in the community in the Pre-intervention and Intervention Phase

When comparisons were made of sociodemographic, pregnancy and infant characteristics, there were no differences between these in the pre**-**intervention and intervention phases except EBR as described in Table 2, nor was there any significant difference (*p* = 0.59) in age of infants between pre**-**intervention and intervention phases. Among pre**-**intervention infants, the EBR was 58% whilst in the intervention infants, this was 78% (*p* = 0.000). Although infants ranging from 0 to 5 months were accessed by a method of community follow up of families in reverse order of discharge from hospital after delivery, this did not provide an equal number of infants in each age category (Fig. 3).

**Table 2:** Characteristics of participats by phase

Background characteristics	Pre-intervention Phase ***n*** = 114 (%)	Intervention Phase ***n*** = 151 (%)	***p*** - value^**a**^
**Sociodemographic characteristics**			
Mother’s educational qualification			
Went to school	112 (98)	145 (96)	0.47
Never went to school	2 (2)	6 (4)	
Mother’s occupation			
Housewife	99 (87)	132 (87)	1.00
Others	15 (13)	19 (13)	
Age of the mother			
< 20 yrs	16 (14)	15 (10)	0.44
20-29 yrs	88(77)	116 (77)	
≥ 30 yrs	10 (9)	19 (13)	
Father’s educational qualification			
Went to school	112 (98)	147 (97)	0.70
Never went to school	2 (2)	4 (3)	
Father’s occupation			
Manual worker	20 (18)	39 (26)	0.14
Non-manual worker	94 (82)	112 (74)	
Type of toilet facility			
Sanitary	109 (96)	146 (97)	0.75
Non-sanitary	5 (4)	5 (3)	
Source of drinking water			
Deep tube well	114 (100)	148 (98)	0.26
Shallow tube well	0 (0)	3 (2)	
Wealth index quintile			
Poorest	3 (3)	7 (5)	0.25
Middle	71 (62)	101 (67)	
Richest	40 (35)	43 (28)	
Mean number of family members	3.97	4.45	0.11
**Pregnancy characteristics**			
Gestational age			
< 37 weeks	27(24)	35 (23)	1.00
≥ 37 weeks	87 (76)	116 (77)	
Mode of delivery			
Vaginal	22 (19)	46 (30)	0.05
Cesarean section	92 (81)	105 (70)	
Outcome of pregnancy			
Single	110 (97)	151 (100)	0.47
Twin	4 (3)	0 (0)	
Parity			
Primipara	61 (54)	73 (48)	0.46
Multipara	53 (46)	78 (52)	
**Infant characteristics**			
Gender of the Infant			
Male	68 (60)	83 (55)	0.45
Female	46 (40)	68 (45)	
Mean birthweight (g)	2784	2892	0.08
**Feeding practice**			
Exclusive Breastfeeding Rate	66 (58)	118 (78)	0.00

^a^*p* - values were based on chi-square test

**Fig. 4:**
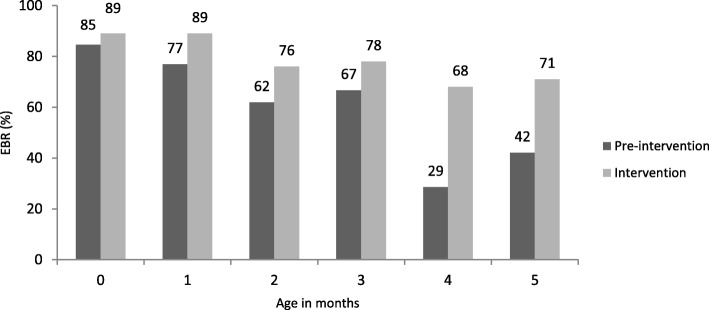
Comparison of EBR between Pre-intervention and Intervention Phases by age of infants

Exclusive breastfeeding remained at a high level in infants delivered in the intervention phase from 89% at less than one month to 71% at five months of age. In contrast, for the pre**-**intervention infants, EBF fell from 85% at less than one month to 42% at five months of age (Fig. 4).

There was no difference in prevalence of diarrhea and pneumonia between pre**-**intervention and intervention phase infants (Table 3).

**Table 3:** Comparison of prevalence of diarrhea and pneumonia among pre-intervention and intervention Infants

Prevalence of diseases	Pre-intervention phase ***n*** = 114 (%)	Intervention phase ***n*** = 151 (%)	***p*** - value ^**a**^
Diarrhea			
Yes	1 (1)	0 (0)	0.43
No	113 (99)	151 (100)	
Pneumonia			
Yes	1 (1)	0 (0)	0.43
No	113 (99)	151 (100)	

^a^*p* - values were based on chi-square test

There was no statistically significant difference between pre**-**intervention and intervention phases in prevalence of wasting (weight**-**for**-**height Z**-**score < **−** 2.00), stunting (height**-**for**-**age Z**-**score < **−** 2.00) or underweight (weight**-**for**-**age Z**-**score < **−** 2.00) (Table 4).

**Table 4:** Assessment of difference in nutritional status between the two phases

Anthropometric parameter	Pre-intervention phase ***n*** = 110 (%)	Intervention phase ***n*** = 148 (%)	***p*** - value^**a**^
Wasted (WHZ^b^ < −2.00)	17 (15)	16 (11)	0.35
Stunted (HAZ^c^ < −2.00)	7 (6)	16 (11)	0.27
Underweight (WAZ^d^ < −2.00)	14 (13)	15 (10)	0.54

^a^*p* - values were based on chi-square test

^b^ Weight-for-height Z-score

^c^ Height-for-age Z-score

^d^ Weight-for-age Z-score

## Discussion

This study has shown that providing breastfeeding training to maternity personnel of a hospital to ensure first hour breastfeeding, assessment and correction of position and attachment, structured counseling of mothers of infants and their relatives on EBF followed by fortnightly mobile phone counseling at home increased EBR in the intervention phase (78%) when compared to the pre**-**intervention phase (58%), both matched for background characteristics (*p* = 0.000). Following discharge home, there was no significant difference in morbidity patterns and nutritional status between the two phases. Anthropometric parameters (wasting, stunting and underweight) were low for both groups compared to national figures [24] and are less likely to be affected by such a short intervention targeted to exclusive breastfeeding. A longer study involving counseling for appropriate complementary feeding in children aged 6–23 months would perhaps show a bigger difference between the two phases with regard to anthropometry.

The method of community follow up of mothers and their young infants, where the last discharged infant was followed up first and the first discharged infant was followed up last, meant that at assessment some mothers had received more counseling phone calls than others. This is acceptable because all studies show that EBF is lower in older infants in this age group, and therefore they would benefit from the increased number of phone calls which is clearly reflected in our study. At five months, large proportions of infants (71%) in the intervention phase were still exclusively breastfed compared to those in the pre**-**intervention phase (42%).

The pre-intervention infants were born between April and June 2010 (summer) and the intervention infants between July and September 2010 (monsoon). Although seasonality has been described as a factor which affects breastfeeding [34, 35], it is unclear in which way this happens. Some studies suggest decrease in the cold season and others in the wet season. Further cohort studies would clarify direction of association with regard to seasonality.

Our study demonstrates that use of mobile phone for follow up of hospital delivered infants for EBF counseling is an effective component of a behavior change communication intervention package. These study results are similar to those that increased breastfeeding duration and exclusivity by providing individual face**-**to**-**face counseling to mother or other caregiver [36–38].

EBF prevalence was much better in our study than in any previous study on proactive telephone breastfeeding support [39–47]. These previous studies were all done in high-income countries with much lower background breastfeeding prevalence than in Bangladesh, where continued breastfeeding rates are 95% at 9–11 and 89% at 20–23 months of age [24]. Furthermore, previous community interventions without telephones in the country have shown that large increases in EBR are possible [48].

### Limitations

This study design has limitations. Firstly, mother**-**infant pairs should have been randomly selected in both intervention and pre**-**intervention phases but budget constraints did not allow for this. This meant that the pre-intervention and intervention phase participants were not matched for season of birth nor for season of community follow up assessment and this may have had an influence on infant feeding. Secondly, the intervention was a package of hospital and community activities. This package included ensuring early initiation of breastfeeding within one hour of birth, followed by repeated face**-**to**-**face support and counseling within 48 h of delivery. Thus, this study could not determine whether any single or a combination of these interventions is most effective. Furthermore, repeated phone calls to mothers in the community may have introduced an element of response bias where mothers felt pressure to respond positively when asked if they are exclusively breastfeeding at home follow-up questionnaire survey. This bias may have been further compounded by the same RAs both providing phone counseling as well as collecting research data. Future investigators may overcome this by separating these two functions.

## Conclusions

This intervention package of multiple components including phone follow up for hospital delivered mothers to sustain EBF in the community led to increased EBR. This has large implications in a country where increasingly more women (50% in 2017–18) are delivering in health facilities and there were 160 million mobile phone users in March 2019. Scaling up this intervention in existing baby friendly maternity facilities would lead to national increases in EBR and ultimately further reduction in child mortality. Further intervention studies are warranted to determine whether the promotion of appropriate complementary feeding in 6–23 month old children improves nutritional status.

## Data Availability

The datasets used and/or analyzed during the current study are available from the corresponding author on reasonable request.

## Abbreviations

BFHI: Baby-Friendly Hospital Initiative
CWCH: Centre for Woman and Child Health
EBF: Exclusive Breastfeeding
EBR: Exclusive Breastfeeding Rate
IYCF: Infant and Young Child Feeding
NGO: Non-Government Organization
OB/GYN: Obstetrician/Gynecologist
RA: Research Assistant
RN: Registered Nurses
